# Impact of order set implementation on appropriate treatment of community-acquired pneumonia (CAP)

**DOI:** 10.1017/ash.2024.467

**Published:** 2025-01-09

**Authors:** Han Pham, Michell J. Stein, Lacy J. Worden

**Affiliations:** Ascension Borgess Hospital, Kalamazoo, MI, USA

## Abstract

**Objective:**

This study aimed to evaluate appropriate antimicrobial prescribing after implementing a pneumonia order set within a community teaching hospital.

**Design:**

Retrospective chart review study.

**Setting:**

450-bed community teaching hospital.

**Participants:**

Patients who are 18 years of age or older admitted for treatment of community-acquired pneumonia (CAP) between October 1, 2021, and August 1, 2023.

**Methods:**

This retrospective cohort study aimed to evaluate a composite endpoint of appropriate empiric antimicrobial selection, dosing, and duration in accordance with the national guidelines after the implementation of a CAP order set. Secondary outcomes included comparing hospital length of stay (LOS), readmission rates, mortality rates, and *Clostridium difficile* infection rates.

**Results:**

A total of 236 patients were included (118 patients per group). Significantly more patients in the post-implementation group received guideline-concordant therapy for CAP (5.9% vs 35.6%, *P* < .001). Results were heavily influenced by improvements in appropriate durations of therapy (pre: 6.8% vs post: 39.9%, *P* < .001). There were no significant differences observed for LOS, 30-day readmission rates, *C. difficile* infections within 30 days, or mortality rates between groups. The order set was utilized in 66.1% of patients included in the post-implementation group.

**Conclusions:**

Implementing an order set significantly improved inpatient antibiotic prescribing for CAP with no difference in clinical or safety outcomes. Antibiotic order sets will be a useful tool for antimicrobial stewardship program expansion into other common community-acquired infections.

## Introduction

Community-acquired pneumonia (CAP) is a leading cause of hospitalization and mortality and is an economic burden to the healthcare system. Incidence can reach 14 cases per 1,000 adults and up to 50% of cases require inpatient hospitalization.^1^ Risk of hospitalization for pneumonia is highest among elderly patients and those with multiple comorbidities such as chronic obstructive pulmonary disease, diabetes, cardiovascular disease, or patients who are immunocompromised.^1^ The primary causative pathogens of CAP include *Streptococcus pneumoniae*, *Haemophilus influenzae*, *Moraxella catarrhalis*, and atypical bacterial species, which are commonly susceptible to more narrow-spectrum antibiotics.^2^

The overuse of broad-spectrum antibiotics is the main cause of antibiotic resistance.^3^ Both *Methicillin-resistant Staphylococcus aureus* (MRSA) and multidrug-resistant *Pseudomonas* are classified by the Centers for Disease Control and Prevention as a “serious” threat of concern to human health affecting approximately 324,000 and 33,000 cases each year, respectively.^3^ Optimizing antimicrobial prescribing and stewardship efforts when treating CAP plays an essential role in combating the global threat of antibiotic resistance. In 2007, the Infectious Diseases Society of America (IDSA) and the American Thoracic Society (ATS) jointly published a guideline outlining empiric antibiotic treatment for CAP. Since then, IDSA/ATS revised their guideline in 2019 and highlighted several new changes. Specifically, they recommended abandoning the term “healthcare-associated pneumonia,” which was defined for those patients who had the potential risk factors, including residence in a nursing home and other long-term care facilities, hospitalization for ≥2 days in the last 90 days, receipt of home infusion therapy, chronic dialysis, home wound care, or a family member with a known antibiotic-resistant pathogen. The updated guideline shifted to categorizing CAP with or without risk factors highlighting only previous MRSA or *Pseudomonas aeruginosa* infection or use of parenteral antibiotics within the last 90 days as validated risk factors for needing anti-pseudomonal and/or anti-MRSA antibiotics empirically.^4^ In addition, recent studies have supported shorter courses of therapy for CAP.^5,6^ The guideline emphasized a total duration of 5 days would be appropriate for most patients.

Efforts to increase guideline-concordant antibiotic prescribing into clinical practice through process standardization have been ongoing for decades to help reduce the use of broad-spectrum antibiotics, shorten antimicrobial duration, and decrease the cost of care.^7–10^ There is a lack of published literature evaluating the impact of order sets on overall appropriate antibiotic therapy in the inpatient setting. The purpose of this study is to evaluate appropriate antimicrobial prescribing for CAP after implementing a pneumonia order set within a community teaching hospital.

## Methods

This was a retrospective cohort study conducted at a 450-bed community teaching hospital. Patients were eligible for inclusion if they were ≥18 years of age with a diagnosis of CAP between October 1, 2021, to August 1, 2022 (pre-intervention period) and October 1, 2022, to August 1, 2023 (post-intervention period). Patients were identified using the *International Classification of Diseases, Tenth Revision* (ICD-10) codes indicating pneumonia. Patients were excluded if they had one of the following criteria: diagnosed with nosocomial pneumonia, history of structural lung diseases, positive severe acute respiratory coronavirus virus 2 polymerase chain reaction during hospital admission, had moderate to severe chronic obstructive pulmonary disease (COPD), history of MRSA or *Pseudomonas* within the last 2 years cultured from any site, any prior MRSA or *Pseudomonas* isolated in a respiratory culture, or any respiratory culture that showed resistance to CAP therapy on admission, history of lung transplant or cancer, did not meet diagnostic criteria for CAP, positive viral panel with symptom onset less than 5 days, or had concomitant infections. Criteria for CAP diagnosis were defined by having positive lung imaging plus two or more of the following clinical findings: cough, sputum production, dyspnea or tachypnea, hypoxemia, fever or hypothermia, WBC >10,000 or >15% bands, or WBC <4,000. Eligible patients were randomized and screened for inclusion via chart review until a convenience sample of 118 patients per group was met. Data collected included patient demographics, pneumonia diagnosis criteria, Charlson comorbidity score, allergies, antibiotic regimen, hospital length of stay (LOS), admission, mortality rate, and the utilization of the order set.

The order set recommended a combination of ceftriaxone intravenous push for 5 days and azithromycin by mouth (PO) for 3 days with stop dates in place upon ordering. The order set also contained a comment indicating the total intended duration of therapy, specifically “including doses given in the emergency department.” This comment allowed the pharmacist to adjust per protocol, the total duration on verification to account for any doses administered in the emergency department. If patients had an allergic reaction to macrolides, the option to replace azithromycin with doxycycline in combination with ceftriaxone was recommended. Levofloxacin PO was reserved for patients with confirmed type-1 allergy or immunologic reaction to penicillin and cephalosporin antibiotics. The CAP order set was presented and approved by the Pharmacy and Therapeutics Committee. It was then incorporated into the electronic health record that could be easily found by prescribers by searching CAP or pneumonia. Additional support for compliance with guideline recommendations and raising awareness about this order set were provided by the antimicrobial stewardship team and pharmacy staff through prospective audit and feedback as standard practice throughout the study period. Our lead hospitalist also took an active role in encouraging provider use of the CAP order set at provider meetings to help provide a multidisciplinary approach. There was a 3-month “washout” period from the time the order set was implemented to the time the data for the post-intervention group was collected.

The primary objective of this study was a composite outcome of appropriate empiric antimicrobial selection, dosing, and duration in accordance with the national guidelines. Secondary outcomes included hospital LOS, readmission rates, mortality rates, and *Clostridium difficile* infection rates. An exploratory outcome was pre-determined to evaluate pharmacists’ intervention on the duration of therapy by evaluating pharmacist per protocol or telephone/verbal with readback antibiotic order adjustments and utilization of the order set to prescribe antibiotics. The antibiotic regimen was obtained via chart review.

For nominal variables, the χ^2^ test and Fisher’s exact test, as appropriate, were used, and results were reported as percentages with *P* values. For interval variables, the *t* test and Mann-Whitney test based on the distribution of the data were used. All analysis was evaluated using SPSS statistical software version 22 using an alpha level of 0.05 such that results yielding *P* < .05 were considered statistically significant. Previous data reported from recent *Michigan Hospital Medicine Safety Consortium (HMS)* monitoring within our community teaching hospital indicated the percentage of appropriate duration of therapy for CAP was about 35%. The anticipated increase in overall appropriate treatment for CAP in the intervention arm from pre-implementation to post-implementation was 18%. This required at least 236 patients to be evaluated (118 patients in each group) to achieve a power of 80% using a 2-independent-samples proportions test. This study was reviewed and approved by the Ascension Health Institutional Review Board.

## Results

The initial query identified 2,314 patients with CAP within the pre-specified timeframe. A randomized sample of 441 patients was screened until 236 patients met the criteria for inclusion (118 patients in the pre-implementation group and 118 patients in the post-implementation group). Patient demographics were largely balanced between groups (Table 1). Overall, about 58% of patients included were male, the mean duration of symptom onset was 4 days, and the mean Charlson comorbidity index was 2. One noteworthy exception was the average age, older patients were more commonly included in the post-implementation group compared to the pre-implementation group (68 vs 72 years old, *P* = .018). A total of 121 and 75 patients were excluded from the study in the pre- and post-implementation cohorts, respectively. The main reason for exclusion criteria was a diagnosis of nosocomial pneumonia (58 of 196, 29.6%). Other reasons were having concomitant infections (39 of 196, 19.9%), lung transplant or cancer (24 of 196, 12.2%), MRSA or *Pseudomonas* respiratory culture or any prior cultures resistant to CAP therapy (18 of 196, 9.2%), and history of structural lung diseases (16 of 196, 8.2%).

**Table 1.** Patient baseline characteristics

Variables	Pre-group	Post-group	*P* value
Sex, male, n (%)	55 (46.6)	61 (51.7)	.435
Age, year, mean (±SD)	68 (±16.1)	72 (±14.3)	.018
BMI, n (5)			.871
Underweight	6 (5.1)	3 (2.5)	
Normal	36 (30.5)	32 (27.1)	
Overweight	26 (22.0)	31 (26.3)	
Obesity class 1	22 (18.6)	22 (18.6)	
Obesity class 2	11 (9.3)	13 (11.0)	
Obesity class 3	17 (14.4)	17 (14.4)	
Duration of onset, days, mean (±SD)	4.26 (±6.9)	4.69 (±5.5)	.626
Charlson comorbidity index, mean (±SD)	1.92 (±1.6)	2.29 (±2.29)	.07
Beta-lactam allergy, n (%)	23 (19.5)	23 (19.5)	1
Anaphylaxis, n (%)	3 (13.0)	1 (4.3)	.608
Fluoroquinolones allergy, n (%)	0	11 (9.3)	<.001

For the primary outcome, the results showed significantly more patients in the post-implementation group received guideline-concordant therapy for CAP (5.9% vs 35.6%, *P* < .001). Results were heavily influenced by improvements in appropriate durations of therapy (6.8% vs 39.8%, *P* < .001). There wasn’t a significant improvement in antibiotic selection (82.2% vs 89.9%, *P* = .091) or dosing between pre- and post-implementation groups (83.9% vs 88.1%, *P* = .348) (Table 2). Following order set implementation, the appropriate duration significantly increased in beta-lactams (44% vs 70.2%, *P* < .001), macrolides (25.7% vs 59.2%, *P* < .001), and tetracyclines (35.7% vs 72%, *P* < .001) (Table 2). There was a significant decrease in the duration of beta-lactam use (5.9 vs 5.1 days, *P* = .013), macrolide (4.7 vs 3.5 days, *P* < .001), and fluoroquinolones (8.2 vs 4.8 days, *P* = .031) (Table 2). More patients in the pre-group had excessive durations of therapy during admission compared to the post-group (37.3% vs 16.1%, *P* < .001), while both groups had high rates of excessive durations of therapy as a result of discharge prescriptions (55.9% vs 44.1%, *P* = .07). For secondary outcomes, there were no significant differences observed for the mean LOS (4.77 vs 4.53 days, *P* = .403), 30-day readmission rates (11% vs 17.8%, *P* = .193), and 90-day mortality rates (1.7% vs 0.8%, *P* = .561). Ninety-day readmission rate was higher in the post-implementation group (15.3% vs 26.3%, *P* = .037). However, there was no difference when looking at readmission rates related to pneumonia (22.2% vs 12.9%, *P* = .443). This result implies that the 90-day readmission rate was due to other baseline comorbidities or acute illnesses rather than pneumonia. There were no *C. difficile* infections and no hospital deaths recorded in either group (Table 3).

**Table 2.** Composite endpoint of appropriate empiric antimicrobial selection, dosing, and duration

Outcome	Pre-group (N = 118)	Post-group (N = 118)	*P* value
Overall appropriate treatment	7/118 (5.9)	42/118 (35.6)	<.001
Appropriate empiric therapy	97/118 (82.2)	106/118 (89.9)	.091
Appropriate dosing (%)	99/118 (83.9)	104/118 (88.1)	.348
Appropriate duration (%)	8/118 (6.8)	47/118 (39.8)	<.001
Beta-lactam	51/116 (44.0)	80/144 (70.2)	<.001
Macrolide	27/105 (25.7)	58/98 (59.2)	<.001
Tetracycline	5/14 (35.7)	18/25 (72)	.027
Fluoroquinolones	1/7 (14.3)	3/8 (37.5)	.569

**Table 3.** Hospital LOS, readmission rates, mortality rates, and *Clostridium difficile* infection rates

Outcome	Pre-group (N = 118)	Post-group (N = 118)	*P* value
Mean LOS, days	4.77	4.53	.403
30-day readmission, n (%)	13 (11)	21 (17.8)	.193
Pneumonia-related, n (%)	3/13 (23.1)	2/21 (9.5)	.348
90-day readmission, n (%)	18 (15.3)	31 (26.3)	.037
Pneumonia-related, n (%)	4/18 (22.2)	4/31 (12.9)	.443
30-day *C. difficile* infection, n (%)	0 (0)	0 (0)	0 (0)
Hospital mortality, n (%)	0 (0)	0 (0)	0 (0)
90-day mortality, n (%)	2 (1.7)	1 (0.8)	.561

Note. LOS, length of stay.

For exploratory endpoints, the order set was utilized in 66.1% of patients included in the post-implementation group. Additionally, pharmacists were more likely to intervene following the implementation of the order set (22.9% vs 43.2%, *P* < .001).

## Discussion

In this retrospective cohort study, implementing a guideline-concordant order set significantly improved the overall antibiotic prescribing for patients admitted with CAP. Additionally, shorter treatment durations were not associated with worse clinical or safety outcomes. The increase in the overall appropriate treatment of CAP was driven largely by improvement in appropriate durations of therapy.

Previous studies have also used order sets as a way to improve antimicrobial prescribing in various settings. Colmerauer and colleagues sought to evaluate the impact of an order set on broad-spectrum antibiotics used in patients admitted with CAP.^7^ A total of 331 patients in the pre-intervention group and 352 patients in the post-intervention group were included. The authors found the overall duration of broad-spectrum therapy including anti-pseudomonal β-lactams and anti-MRSA antibiotics was reduced from a median of 2 days to 0 days following implementation of the order set (IQR 0–8 vs IQR 0–4, *P* < .001). Krive et al conducted a study to evaluate the impact of computerized physician order entry on inpatient mortality and readmission rates in patients with CAP over a 5-year period (2007–2011)^8^. They found a reduction in 30-day readmission (OR = 1.362; 95% CF 1.015–1.827; *P* = .039) and LOS (4.79 days vs. 4.32 days *P* = .009), despite the low utilization of an order set (9% and 11.3% usage in the pre- and post-intervention cohorts). It is likely that with increased utilization of the order set, the outcomes would continue to improve. Of note, our study observed order set utilization in 66.1% of patients in the post-implementation group, which is significantly higher than previous studies have described. We hypothesize this was likely due to ease of use, collaboration with our lead hospitalist, and prospective audit and feedback by pharmacists when the order set was not used.

Improved antimicrobial prescribing in the emergency department and primary care settings through order set implementation has also shown positive outcomes for other common infectious diseases.^9,10^ Seizt et al evaluated the impact of order sets that were created for cystitis, pyelonephritis, pneumonia, COPD, and cellulitis in the emergency department.^9^ Their study showed patients were more likely to receive appropriate antibiotics (86.4% vs 33.8%, *P* < .001) and have an appropriate duration prescribed (68.2% vs 24.5%, *P* = .0004) when order sets were used. Similarly, Foreman and colleagues sought to evaluate the impact of an order set for urinary tract infections (UTI) and skin and soft tissue infections (SSTI) in an outpatient setting. This study included 260 patients and resulted in significantly improved antibiotic appropriateness from 24.5% to 39.7% (*P* = .008), appropriate drug selection improved from 52.5% to 66.9% (*P* = .018), and duration from 47.5% to 68.6% (*P* = .001).^10^

There were a number of limitations that may have impacted the outcome of this study. First, as with all single-center retrospective studies, we relied heavily on accurate documentation, which may limit external validity. However, the results of our study were similar to previous published studies that have used order set implementation to improve prescribing in other practice settings. Another limitation was antibiotics administered to patients transferred from outside facilities may not have been captured. To limit interpretation discrepancies, all data was collected by a single investigator. Additionally, we had very strict inclusion criteria that aligned with guideline recommendations for narrow spectrum and short antimicrobial duration, which may limit the generalizability of our findings. Furthermore, pharmacists were more likely to intervene on antimicrobial orders in the post-implementation group; therefore, the improvement in appropriate overall therapy for CAP may have been influenced by empowering pharmacists to adjust the total duration of therapy to account for doses administered in the emergency department and educational efforts. However, it is worth noting that in our study, the order set utilization rate was 66.1%, relatively higher compared to other studies, which ranged from 4% to 21%.^7–9^ Lastly, although we saw significant improvement, both pre- and post-groups had overall low rates of appropriate durations of therapy which was largely driven by excessive antimicrobials prescribed at discharge. Future studies would benefit from incorporating antimicrobial transitions of care along with order set implementation to prevent excessive durations of therapy on discharge.

In conclusion, implementing a guideline-concordant order set significantly improved inpatient antibiotic prescribing for CAP with no difference in clinical or safety outcomes. Opportunities to further explore the benefits of order sets in the inpatient setting for antimicrobial stewardship programs into other infections such as SSTI and UTI in the inpatient setting should be explored in the future.
